# The role of epigenetics in personalized medicine: challenges and opportunities

**DOI:** 10.1186/1755-8794-8-S1-S5

**Published:** 2015-01-15

**Authors:** Mahmood Rasool, Arif Malik, Muhammad Imran Naseer, Abdul Manan, Shakeel Ahmed Ansari, Irshad Begum, Mahmood Husain Qazi, Peter Natesan Pushparaj, Adel M Abuzenadah, Mohammed Hussein Al-Qahtani, Mohammad Amjad Kamal, Peter Natesan Pushparaj, Siew Hua Gan

**Affiliations:** 1 Center of Excellence in Genomic Medicine Research (CEGMR), King Abdulaziz University, Jeddah, Saudi Arabia; 2 KACST Technology Innovation Center in Personalized Medicine, King Abdulaziz University, Jeddah, Kingdom of Saudi Arabia; 3 Institute of Molecular Biology and Biotechnology, (IMBB), the University of Lahore, Lahore, Pakistan; 4 Center for Research in Molecular Medicine (CRiMM), The University of Lahore, Pakistan; 5 King Fahd Medical Research Center, King Abdulaziz University, Jeddah, Saudi Arabia; 6 Human Genome Centre, School of Medical Sciences, Universiti Sains Malaysia, 16150 Kubang Kerian, Kelantan, Malaysia

## Abstract

Epigenetic alterations are considered to be very influential in both the normal and disease states of an organism. These alterations include methylation, acetylation, phosphorylation, and ubiquitylation of DNA and histone proteins (nucleosomes) as well as chromatin remodeling. Many diseases, such as cancers and neurodegenerative disorders, are often associated with epigenetic alterations. DNA methylation is one important modification that leads to disease. Standard therapies are given to patients; however, few patients respond to these drugs, because of various molecular alterations in their cells, which may be partially due to genetic heterogeneity and epigenetic alterations. To realize the promise of personalized medicine, both genetic and epigenetic diagnostic testing will be required. This review will discuss the advances that have been made as well as the challenges for the future.

## Introduction

**Figure 1:**
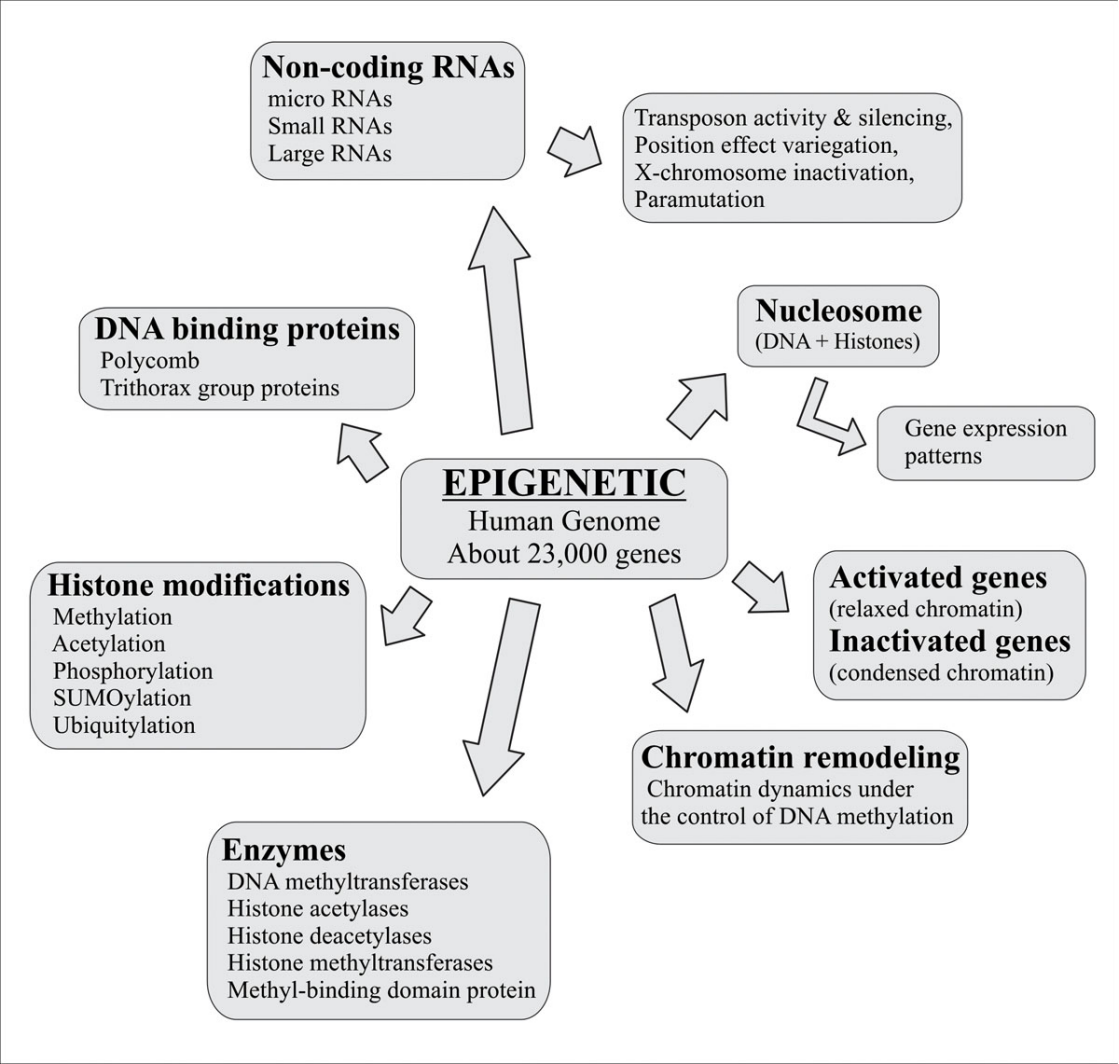
Epigenetic alterations in biological systems

Modifications in gene expression that are independent of the DNA sequence of a gene are called epigenetic alterations. These alterations may contribute to epigenetic inheritance and epigenetic carcinogenesis or any other disease related to alterations in an organism. The epigenetic modifications and/or information are propagated transgenerationally to daughter cells through multiple somatic cell divisions (figure 1). An organism’s genome can be modified by various chemical compounds or species in the biological system leading to changes in gene expression; these modifications are called the epigenome. Changes in the internal and external environment of a biological system, such as oxidative and nitrosative stress as well as nutritional changes, may lead to epigenetic alterations [1,2]. An organism’s genotype has the ability to exhibit phenotypic variation caused by the influence of multiple environmental factors. This ability is called plasticity, and the most favorable form of plasticity occurs during development to increase the survival rate and reproductive success of an organism [3].

Modifications in gene expression are controlled by these fundamental epigenetic mechanisms (figure 1): DNA methylation [4], histone modifications [4,5], chromatin remodeling and microRNAs that act as regulatory molecules [6]. These mechanisms regulate gene expression as well as various cellular and biological functions related to homeostasis, allostasis and disease. The phenotypic variations in humans caused by epigenetic modifications may lead to various diseases [7-9] including bone and skin diseases associated with autoimmune disorders [10], neurodegenerative diseases such as schizophrenia [11,12] and cancer [13-16]. Therefore, traditional therapies may be ineffective to treat patients with epigenetic causes of disease. As a result, researchers are inclined to find patient-specific treatments for these patients, which are referred to as personalized or genomic medicines.

## Epigenetic modifications

DNA methylation is considered to be one of the most important modifications leading to disease. Multiple processes, including gene expression, X-chromosome inactivation, imprinting, chromatin organization and other biological processes are controlled by DNA methylation [4]. The addition of a methyl group (-CH_3_) to cytosine frequently occurs at gene promoter regions with CpG islands, which are regions of large repetitive CpG dinucleotides occupying 60% of the promoter region [17]. Methylation of CpG dinucleotide(s) has been associated with disease states including cancer [18]. The enzymes responsible for DNA methylation are the DNA methyltransferases (DNMTs), which are categorized into five classes based on their specific enzymatic and physiological functions [4]. Another example of epigenetic modification is the modification of histones [4,5], which occur through various nuclear, enzyme-catalyzed mechanisms that lead to modifications including methylation and acetylation of arginine and lysine [19], phosphorylation of threonine and serine, sumoylation of lysine, ubiquitination and ADP-ribosylation [6]. Multiple diseases such as Parkinson’s disease, Angelman syndrome and mental retardation have been associated with ubiquitination (table 1). The acetylation of histone proteins at various amino acid residues is regulated by histone acetyltransferases (HATs) and histone deacetylases (HDACs) [18] (figure 1). The process of methylation occurs through the transfer of a methyl group to a histone from adenosyl methionine (AdoMet), and S-adenosylhomocysteine (AdoHcy) inhibits the action of DNMTs. AdoHcy hydrolase can hydrolyze AdoHcy into adenosine and homocysteine, and therefore, could be employed as a therapeutic agent for epigenetic diseases. Catalytic ATPases are involved in the energy driven alterations of nucleosome positioning and DNA-histone associations during the process of chromatin remodeling [[20], table 1].

**Table 1:** Multiple diseases related to ubiquitination.

Sr. No.	Gene	Encodes for	Disorder	Citation
**1**	*Parkin*	E3 ubiquitin ligase	Parkinson’s disease (autosomal recess)	[21]

**2**	*uchl1*	Ubiquitin C-terminal hydrolase (UCH-L1)	Parkinson’s disease (autosomal recess)	[21]

**3**	*E6-AP*	Ubiquitin ligase E6-AP (UBE3A)	Angelman syndrome	[22]

**4**	Single point mutation in *HUWE/Mule/ ARF-BP*	Ubiquitin ligase HUWE/Mule/ ARF-BP	Mental retardation, X-linked, syndromic Turner type (MRXST)	[23,24]

**5**	*Ataxin-3*	Deubiquitinating enzyme, ataxin-3	Familial amyotrophic lateral sclerosis, Machado-Joseph disease/ spinocerebellar ataxia type-3	[25]

**6**	Hippel Lindau *vhl*	E3-ubiquitin ligase	Pheochromocytoma (PCC)	[26]

**7**	*cyld*	Deubiquitinase CYLD	Turban tumor syndrome (cylindromatosis)	[27]

**8**	Aberrant expression/ mutations	E3 ubiquitin ligase/ deubiquitinating enzymes (DUBs)	Diverse types of cancer	[28-30]

## Genetic testing/screening

The clinical utility of a medical test is determined by the ability of the test results to alter the decisions of physicians or the types of health care used to treat the disease. The diagnosis of a disease is based on signs and symptoms that may be indicative of several disorders in a biological system. At present, it is possible to determine the prognosis and diagnosis of any disorder through genetic testing or screening for disease-specific mutations. A large number of molecular biomarkers related to gene mutations can be identified through genomic studies. The results of prognostic and diagnostic tests using genomic data or DNA are used by health care professionals to diagnose disorders or diseases, to assess the risk of disease in an individual, to establish appropriate dosage for an individual based on variations in metabolism and to determine whether an individual will benefit from a particular drug intervention for disease management.

On the other hand, personalized medicine is the application of an individual’s personal genetic profile to predict disease, prevent disease through medical interventions, and make decisions about lifestyle and disease management based on the needs of each individual patient. Moreover, genetic screening is important for the personalization of treatment for a patient.

## Epigenetics and personalized/genomic medicine

The study of the genome and its related information can shed light on various questions associated with the health and disease of an individual. Whole-genome DNA sequence information is now accessible due to the completion of the Human Genome Project (HGP). Specific drugs must be used for those patients who are not responding to traditional medicines as expected and for whom the rate of successful disease management is very low. Genomic or personalized medicines are given to patients after collecting genomic information and associated data such as the levels of RNA, proteins and various metabolites that are crucial factors in medical decision making for personalized medicine [31].

Genomic approaches such as the identification of DNA sequence variations, transcriptomics, proteomics and metabolomics are useful for precise disease management and prediction [32]. These approaches are useful tools that bridge epigenetics and personalized medicine: the human genome sequence (genomics) includes 10-15 million single nucleotide polymorphisms (SNPs) and copy number variants (CNVs); gene expression profiles (transcriptomics) consist of approximately 25,000 gene transcripts; the proteome (proteomics) includes approximately 100,000 specific protein products; and the metabolome (metabolomics) is a metabolic profile of 1000 to 10,000 metabolites [32]. Moreover, the information from an individual genome sequence and the associated expressed biomarkers also are imperative to achieve personalized and genomic therapies [33].

For a chronic disease, as traditional medicine or treatments may be ineffective for patients, the risk of disease may be inherited and reflect the patient’s genomic background. While observing a patient from a healthy state to a diseased state, genomic applications can be used at various crucial checkpoints to personalize the individual’s health care [34,35].

## Pharmacogenomics and personalized medicines

Pharmacogenomics deal with various biological factors related to drug metabolism including drug transporters, the contribution of receptors and drug metabolizing enzymes with polymorphisms that affect the drug response in a variety of diseases [36,37]; all of these parameters are under epigenetic control.

Pharmacogenomics help us to understand the idea of the precise and accurate drug for a respective patient at the accurate concentration and time. Moreover, it negates the concept of “one drug fits all.” As far as multiple drug responses are concerned, various factors such as nutrition, age, body weight, sex, genetic behavior, infections, co-medications and organ function are important considerations that are unavoidable during the course of treatment for a disease. Furthermore, the integration of relevant data associated with medical informatics and personalized medicines is highly targeted for the management of a disorder.

**Figure 2:**
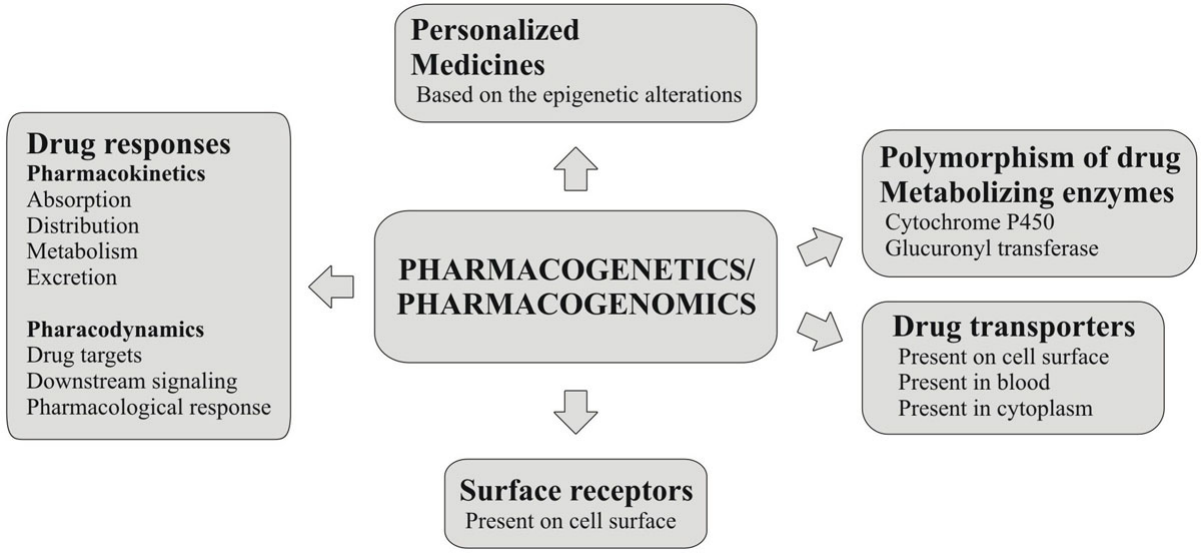
Roles of pharmacogenomics and pharmacogenetics in disease treatment and personalized medicine

To understand variable drug responses (traditional and/or personalized medicines), pharmacokinetics (PK) and pharmacodynamics (PD) are highly useful. These two disciplines integrate quantitative measurements of drug exposure and effect (figure 2). Pharmacokinetics data are associated with drug exposure and the monitoring of drug levels, providing a platform to analyze the phenotypic markers (epigenetic markers) useful for personalized medicine. Variability in drug response is often linked to alterations or mutations in the drug metabolizing enzymes cytochrome P_450_ and glucuronyl transferase, encoded by the polymorphic genes of the *CYP450* family [38], as well as drug transporters encoded by several hundred genes [39]. Microarray technology can be used to detect the 29 known variants of two important *CYP450* genes, CYP2D6 and CYP2C19; these genes affect the metabolism of 25% of all prescribed drugs [40].

Moreover, drug receptors are also encoded by polymorphic genes [39] and mutations in receptors, such as the receptor tyrosine kinases, have been linked to various cancers and neurodegenerative diseases [41-44]. For example, over-expression of ErbB2 (v-erb-b2 avian erythroblastic leukemia viral oncogene homolog 2) in breast cancer is treated with trastuzumab [43], the BCR/ABL fusion protein is highly sensitive to imatinib in the case of leukemia [41] and activating mutations of epidermal growth factor receptor (EGFR) seem to correlate with the responsiveness to gefitinib [42]. Hence, genotyping becomes very important for researchers to better understand a disease, its management and drug effects within the practice of personalized medicine. Once functional polymorphisms and genetic variability have been experimentally established for well-studied genes, this knowledge can be applied to future clinical studies.

Genetic variants are transcribed into mRNA and can affect its processing, including mRNA stability and alternative splicing. Studies have revealed that alternative splicing occurs in approximately 35-59% of all human genes [45]. Allelic expression has been analyzed based on mRNA expression and revealed that the catechol-O-methyltransferase gene (COMT) is a susceptibility gene for schizophrenia that is downregulated in the autopsy brain tissues of patients [46]. Epigenetic changes affect various disorders, including cancer and neurodegenerative diseases and their treatment outcomes [47-52]. Epigenetic and drug management data from disease patients are useful for the personalization of medicine.

Cancer classification is based on the histological analysis of tissues and/or cells. In the case of some tumors, such as leukemia and breast cancer, molecular biomarkers are used. Moreover, the mRNA expression profiles obtained through microarray analysis also contribute to the identification and classification of many cancers, such as colon, hematological and early stage breast cancers [53-59]. In the case of cancer, targeted therapy is based on gene alterations in specific cellular pathways, which aid the application of genomic medicine [42,60].

Targeted cancer therapy involves tumor cell-specific treatments including monoclonal antibodies and small molecule inhibitors that are less toxic in their mode of action. This therapeutic strategy has opened new possibilities for cancer management. Several molecular targets and signaling pathways such as the aurora kinases, the FOXO-FOXM1 axis [61] and PI3K/mTOR signaling [62] are involved in human cancers. For example, the small molecule VX-680 shows an inhibitory effect and induces cell death in leukemic cells with a specific aurora expression profile by increasing the Bax/Bcl-2 ratio, which induces apoptosis in acute myeloid leukemia with high aurora-A expression [63].

Similarly, forkhead transcription factors (FOXO and FOXM1) play crucial roles in cell division, differentiation, angiogenesis, apoptosis, DNA repair and tissue homeostasis. Moreover, the FOXO-FOXM1 axis plays an important regulatory role in drug resistance and tumorigenesis [61].

## Current personalized medicines

Some patients with late-stage non-small cell lung cancer (NSCLC) have rearrangements in the anaplastic lymphoma kinase (*ALK*) gene, and conventional cancer therapies are ineffective for these patients (table 2). Hence, crizotinib (Xalkori^®^), an anti-cancer drug that inhibits ROS1 (c-ros oncogene 1) and ALK, is used for the 5% of patients that will respond to this drug because they have a chromosomal rearrangement that produces a gene fusion (ALK and EML4, echinoderm microtubule-associated protein-like 4) that results in carcinogenesis. Genetic tests are recommended for multiple diseases that could be treated with personalized medicine; there are approximately 1,600 molecular diagnostic tests available that target multiple disorders [64]. Many patients do not respond to first-line therapies, and studies have shown that this lack of response is due to differences in the genes that encode drug targets, transporters and metabolizing enzymes such as cytochrome P_450_ and glucuronyl transferase [65-67].

For various types of cancers, molecular diagnoses are available that assist physicians in improving disease management and increasing the chance of patient survival (table 2). For example, melanoma cases are classified based on the results of the *BRAF* genetic test. Moreover, non-small cell lung cancers can test positive for *ALK* and *BRAF* mutations, which is useful for targeting the *ALK* and *BRAF* gene alterations during the course of molecular treatment [68].

Different cancers exhibit different rates of genetic mutations that drive carcinogenesis: melanoma has the highest rate of genetic mutation (73%), while thyroid cancer has the second highest (56%). Following these cancers in driver mutation prevalence are lung cancer at 41%, gynecological cancers at 31% andgastrointestinal cancers at 25% and both ovarian and head and neck cancers at 21% [68]. For example, patients with mutations in the *BRCA1* or *BRCA2* genes have a 36 to 85% chance of developing breast cancer compared with a 13% risk among the general female population [69,70]. In breast cancer cases, approximately 30% of cancers exhibit over-expression of the cell surface protein known as human epidermal growth factor receptor 2 (HER2), and the standard therapy is not effective in these HER2-overexpressing patients (table 2). However, the antibody drug trastuzumab can decrease the recurrence of HER2-positive tumors by 52% when combined with chemotherapy, a response greater than that for chemotherapy alone [71,72]. While patients with metastatic colon cancer with a *KRAS* mutation could be treated with cetuximab (Erbitux^®^) and panitumumab (Vectibix^®^), it is recommended that only patients with a normal *KRAS* gene be treated with these drugs in combination with chemotherapy [73,74].

## Conclusion

A given genotype has the ability to confer a variety of phenotypes in the presence of different environmental factors; this ability is called plasticity. Modifications in gene expression are controlled by fundamental epigenetic mechanisms including DNA methylation, histone modifications, chromatin remodeling and microRNAs that act as regulatory molecules. Various tools are used to identify phenotypic or epigenetic alterations in biological systems. Such environmentally influenced alterations may lead to several disorders and patients with epigenetic alterations and their associated disorders do not respond to conventional therapy. Therefore, drugs used for personalized medicine can be used to manage these disorders based on an individual’s personal genomic profile. Many of the drugs used for personalized medicine have been approved by the FDA. However, various challenges exist for the scientists and researchers studying genomic alterations and their phenotypic expression given that each patient is unique.

## List of abbreviations

DNA: deoxyribonucleic acid; -CH_3_: methyl group; DNMTs: Methyltransferases; HAT: histone acetyltransferases; HDAC: histone deacetylases; AdoMet: adenosyl methionine; AdoHcy: Adenosylhomocysteine; HGP: Human Genome Project; SNPs: single nucleotide polymorphisms; CNVs: copy number variants; PK: Pharmacokinetics; PD: Pharmacodynamics; CYP450: cytochrome P_450_; EGFR: epidermal growth factor receptor; ErbB2: v-erb-b2 avian erythroblastic leukemia viral oncogene homolog 2; COMT: catechol-O-methyltransferase; FOXO, FOXM1: forkhead transcription factors; NSCLC: non-small cell lung cancer; *ALK:* anaplastic lymphoma kinase; *EML4:* echinoderm microtubule-associated protein-like 4; *BRAF:* v-RAF murine sarcoma viral oncogene homolog B1; HER2: human epidermal growth factor receptor 2.

## Conflict of interest

The authors declare that they have no competing interests.

## Authors’ Contribution

Rasool M, Malik A, Naseer MI, Manan A, searched the literature using various internet tools and wrote the manuscript. Ansari SA, Begum I, Qazi MH, Pushparaj PN,analyzed the data and reviewed the manuscript critically. Abuzenadah AM, Kamal MA, Gan SH, significantly contributed in final editing to improve the quality of the manuscript. Al-Qahtani MH approved the final manuscript for publication.

## Declarations

This article has been published as part of *BMC Medical Genomics* Volume 8 Supplement 1, 2015: Selected articles from the 2nd International Genomic Medical Conference (IGMC 2013): Medical Genomics. The full contents of the supplement are available online at http://www.biomedcentral.com/bmcmedgenomics/supplements/8/S1
